# Supplementary material for: Inverse reconstruction of model cells: Extracting structural and molecular insights through infrared spectroscopic cytology
Source: PLoS One. 2025 May 6;20(5):e0320697. doi: 10.1371/journal.pone.0320697 (PMC12054921; doi:10.1371/journal.pone.0320697)
Supplement: Attachment — Submitted filename: Response to Reviewers.pdf [file pone.0320697.s001.pdf]

Dear Editor

We thank the reviewers for their professional and very valuable comments and questions. By following their advice, answering all of their questions, and implementing all of their suggestions, we have prepared and resubmitted a revised version of the manuscript.

We believe that answering the reviewers' questions in full and revising our manuscript accordingly (see "Actions taken") strengthened our manuscript significantly and made it easier to read for the Journal's audience.

In the following, we provide our answers to the comments by the two reviewers, point-by-point, and explain in detail how our manuscript has been revised.

Reviewer 1:

At first, I want to declare that I am not professional in the area of complex permittivity measured by the infrared spectroscopy. This work by Proity Nayeeb Akbar and Reinhold Blümel describes the inverse reconstruction of complex permittivity through infrared spectroscopy. They also discussed the fits between simulated extinction efficiencies to the experimental extinction efficiencies. In total, they have compared the complex permittivity and extinction efficiencies of six materials named PMMA, PC, PDMS, PEI, PET and PS respectively. It was well written and sounds good. In view of such interesting results, Plos one is the potential journal to publish this work. Prior to further considerations, the following issues should be considered:

1. The authors listed the experimental permitivities (real and imaginary parts) and extinction spectrum together. However, I am not sure what are their relations between equation 3 and equations 10-11.

Answer:
We thank the referee for this excellent question. Indeed, we had not explained in our the paper (a) why in Figures 3 and 4 we show the experimental permitivities (real and imaginary parts) and extinction spectrum together and (b) what the relationship is between the permittivities (equations 10-11) and the extinction spectrum (equation 3).

To answer part (a) of the referee's question: In thin-film infrared spectroscopy there is a nearly one-to-one relationship between peaks in the imaginary part of the permittivity and peaks in the extinction spectrum. Because of the scattering contributions of finite-size samples, such as cells or spheres, scattering contributions distort this nearly one-to-one correspondence, but, as shown in Figures 3 and 4, vestiges of this relationship remain. Therefore, to bring out this approximate relationship, we show the permittivities and the extinction spectra together in Figures 3 and 4. This correlation, however, as illustrated by Figures 3 and 4, is too vague to form the basis of a simple reconstruction method. The full inverse, numerical method, as outlined in our paper, is required.

To answer part (b) of the referee's question: The referee correctly identifies the central point of our paper, namely the difficulty in determining the permittivities from the extinction spectrum: No simple analytical connection exists between the permittivities (equations 10-11) and the extinction spectrum (equation 3). While no simple formula exists that connects the permittivities to the extinction spectra, at least this direction of their connection is straightforward and can be established efficiently using numerical methods. This direction is what we call the forward model in our paper. The reverse direction, i.e., finding the complex permittivities from the extinction spectra, is much more complicated and finding and presenting an algorithm that accomplishes this inverse procedure, and accomplishes it well with excellent accuracy, is the central point of our paper.

Action taken:

Acting on the referee's comment part (a), we inserted a paragraph (see blue text at the end of Section 3.2.1) that now explains why we show both the permittivities and the extinction efficiencies in Figures 3 and 4. This new text is along the lines of our answer to part (a) of the reviewer's question.

Acting on the referee's comment part (b), we now mention the absence of a direct analytical connection between $Q_{\mathrm{ext}}(\tilde{\nu})$ and $\tilde{\epsilon}_r(\tilde{\nu})$ explicitly in our paper (see blue text at the end of the revised Section 2.2). This also gave us an opportunity to put our inverse-scattering method into the context of trapdoor problems, which many readers might be familiar with, and which further explains why the reconstructions performed in our paper are so much more difficult than the forward problem, i.e., computing $Q_{\mathrm{ext}}(\tilde{\nu})$ from $\tilde{\epsilon}_r(\tilde{\nu})$. We thank the reviewer for having given us this opportunity for further putting our method into context.

2. In the manuscript, the authors listed Figure 5, however, they did not discuss this figure in main text. Please check the 3.3 part dielectric functions versus anti-symmetrized lorentzians.

Answer:

We thank the reviewer for pointing this out to us. There was a typographical error in the LATEX file of our paper, which caused Figure 6 to be called twice, while Figure 5 was not called at all. So, in fact, Figure 5 was indeed already discussed in the original version of our paper, but because of the typographical error, Figure 6, instead of Figure 5, was called in the context of the Figure 5 discussion.

Action taken:

We corrected the typographical error in the LATEX file. As a result, Figure 5 is now called in the text and is discussed in the text. Correcting the typographical error also automatically reordered and renamed Figures 5 and 6 correctly in the sequence in which they are called. In addition, we added a paragraph in Section 3.3 that now discusses Figure 6 in some more detail and adds a comparison between Figures 5 and 6.

3. In the Figure 5 and 6, why the authors applied the constructed fitting of 17 optimized dielectric/anti-symmetric Lorentzian functions?

Answer:
We thank the reviewer for pointing out this important point. Indeed, we had not even explained why we settled on 17 functions for the earlier Figure 1. This was done since, as a result of extensive fitting simulations, we found that a minimum of 17 basis functions was sufficient to obtain good fitting accuracy. Fewer basis functions produced fits that are not so good. More functions improve the fits, but not significantly. Therefore, 17 turned out to be the minimal choice of the number of basis functions, but leaving the door open to run reconstructions with more basis functions.

Action taken:
We now discuss the choice of the 17 functions in detail in our paper. We also mention that we performed additional reconstructions with 18, 19, ..., 25 basis functions that confirmed our observations with 17 basis functions (see added blue text in Section 3.1).

Reviewer 2:

Well conceived manuscript. It would be good if recent studies (references/ work done) would be added in the manuscript This study can add in the existing knowledge related infrared microspectroscopy. These findings are significant as they not only improve the accuracy of the reconstructions, but also advance the use of IR microspectroscopy for application in clinical cytology, where precise molecular analysis is vital in diagnosing and monitoring diseases at the cellular level.

Answer:
We agree with reviewer 2 that the reader might appreciate some recent studies and references.

Action taken:
Following the reviewer's suggestion, we added a paragraph in the Introduction of our manuscript discussing several recent works and putting our method into the context of existing methods (see added blue text in the Introduction section). In this paragraph we have added the following new references:

[R1] Hai Liu, Yu Song, Tingting Liu, Jiawen Li, Guixiang Wang, and Li Zhao. Blind infrared spectral signal reconstruction with improved total variation constraint. In 5th International Conference on Computer Information and Big Data Applications (CIBDA 2024), April 26–28, 2024, Wuhan, China. ACM, New York, NY, USA, 6 pages. https://doi.org/10.1145/ 3671151.3671276.

[R2] Josef Brandt, Karin Mattsson, and Martin Hassellov. Deep Learning for Reconstructing Low-Quality FTIR and Raman Spectra - A Case Study in Microplastic Analyses. Anal. Chem. 2021, 93, 16360-16368.

[R3] Eirik Almklov Magnussen, Boris Zimmermann, Uladzislau Blazhko, Simona Dzurendova, Benjamin Dupuy–Galet, Dana Byrtusova, Florian Muthreich, Valeria Tafintseva, Kristian Hovde Liland, Kristin Tøndel, Volha Shapaval, Achim Kohler. Deep learning-enabled Inference of 3D molecular absorption distribution of biological cells from IR spectra. Commun. Chem. 2022, 5 (1), 175. https://doi.org/10.1038/s42004-022-00792-3.
