# Supplementary material for: Inverse reconstruction of model cells: Extracting structural and molecular insights through infrared spectroscopic cytology
Source: PLoS One. 2025 May 6;20(5):e0320697. doi: 10.1371/journal.pone.0320697 (PMC12054921; doi:10.1371/journal.pone.0320697)
Supplement: Attachment — Submitted filename: Response letter to reviewers.pdf [file pone.0320697.s003.pdf]

Dear Editor

We would like to express our sincere gratitude to the Reviewers for their comments and questions. Reviewers 1 and 2 now recommend our paper for publication. Reviewer 3 had some minor comments, which we have carefully addressed and made the requested revisions in the manuscript. In particular, the references are now in square brackets and occur before the punctuation marks. We also corrected some minor typos in the text and in the equations.

We believe that answering the Reviewers' questions in full and revising our manuscript accordingly (see "Actions taken") strengthened our manuscript significantly and made it easier to read for the Journal's audience.

In the following, we provide our answers to the remaining comments of Reviewer 3, point-by-point, and explain in detail how our manuscript has been revised.

Reviewer 3:

1. The abstract is not as strong as it could be. While it includes critical components such as background, methodology, and significance, it can be improved for better clarity, readability, and focus.

Answer:
We thank the Reviewer for this valuable comment. While we believe the abstract is already well focused, we agree that it can be slightly refined for improved readability and flow, making it more accessible to non-experts in the field.

Action taken:
We improved clarity by simplifying some of the phrases and making them less dense. We improved readability by breaking up long sentences and minimizing technical jargon. We also improved the strength of the abstract by a comment explicitly comparing our method to literature methods.

2. According to PLOS ONE's submission guidelines, references should be cited in the text using square brackets, placed immediately after the relevant information and before any punctuation marks. Please check this.

Answer:
We thank the Reviewer for pointing this out to us.

Action taken:
In accordance with the Reviewer's suggestion, we have updated the reference style in the manuscript to align with the PLOS ONE submission guidelines.

3. What does "Opt" stand for?

Answer:
In response, we would like to clarify that the word "opt" in the sentence "directing most clinical environments to opt for commercial bench-top infrared

instrumentation" in the Introduction is a standard English verb, which, according to the online Webster's Dictionary of English, means "to make a choice" or "to decide in favor of something" from a range of possibilities. We hope this clears up any confusion.

4. Analysis and Interpretation: Figures 1-4 are not well interpreted and compared with the literature. Additionally, Figures 1-4 are not visible. Please make the necessary revisions.

Answer:
Concerning interpretation of our figures, Figures 1 and 2 are explained in Section 3.1: "Quality of the Basis," Figure 3 is discussed in Section 3.2.1: "Case I: Reconstruction with no prior knowledge of the material's absorption spectrum," and Figure 4 is covered in Section 3.2.2: "Case II: Reconstruction with some prior knowledge of the material's absorption spectrum."

Concerning comparison with the literature, we agree with the Reviewer that such comparisons are useful.

However, concerning Figures 1 and 2, comparison with the literature is not possible because, as pointed out in our paper, our basis function inverse scattering method is new and therefore literature results do not exist. In addition, the literature does not provide quantitative measures of fit quality, which is another new feature of our methods. Therefore, in connection with Figures 1 and 2 comparisons with the literature cannot be done since comparative data do not exist in the literature.

Concerning Figures 3 and 4, we did indeed follow the Reviewer's suggestion. We now discuss Figures 3 and 4, in particular their quality of fit, in comparison with the literature (see blue text at the end of Section 3.2.2.).

In this context, we mention that a brief comparison of our method with the literature is also provided in the Introduction.

We are unsure what the Reviewer means by "not visible", as Figures 1-4 are clearly visible. We note that the other two Reviewers had no problems in this respect with Figures 1-4.

5. The abstract needs revision.

Answer:
See our answer to the Reviewer's point 1 above.
