# Supplementary material for: Inverse reconstruction of model cells: Extracting structural and molecular insights through infrared spectroscopic cytology
Source: PLoS One. 2025 May 6;20(5):e0320697. doi: 10.1371/journal.pone.0320697 (PMC12054921; doi:10.1371/journal.pone.0320697)
Supplement: Attachment — Submitted filename: Comment on manuscript PONE-D-24-54445R1.docx [file pone.0320697.s002.docx]

Comments on manuscript PONE-D-24-54445R1, entitled "Inverse Reconstruction of Model Cells: Extracting Structural and Molecular Insights through Infrared Spectroscopic Cytology"

The study develops an advanced inverse scattering algorithm for accurately reconstructing the dielectric properties of single biological cells using infrared (IR) microspectroscopy. By analyzing spherical model cells filled with six organic test substances, the method successfully extracts complex permittivities, improving the accuracy of molecular analysis. The results show that using dielectric functions for reconstruction outperforms other methods, advancing the potential of IR microspectroscopy for precise cellular diagnostics, especially in detecting malignancies and monitoring diseases, with no prior knowledge of spectroscopic principles required. This technique holds promise for enhancing clinical cytology and disease diagnosis. Prior to publication, the following minor revisions are required:

1. The abstract is not as strong as it could be. While it includes critical components such as background, methodology, and significance, it can be improved for better clarity, readability, and focus.
2. According to PLOS ONE's submission guidelines, references should be cited in the text using square brackets, placed immediately after the relevant information and before any punctuation marks. Please check this.
3. What does "Opt" stand for?
4. Analysis and Interpretation: Figures 1-4 are not well interpreted and compared with the literature. Additionally, Figures 1-4 are not visible. Please make the necessary revisions.
5. The abstract needs revision.
